# Interventions for bleaching-induced tooth sensitivity: an umbrella review

**DOI:** 10.3389/froh.2026.1927686

**Published:** 2026-09-21

**Authors:** Madina A. Kurmanalina, Zhanar G. Tankibayeva, Togzhan B. Sagimbayeva, Elnara K. Ismagulova, Assylzhan M. Messova, Amin Tamadon

**Affiliations:** 1 School of Dentistry, West Kazakhstan Marat Ospanov Medical University, Aktobe, Kazakhstan; 2 School of Dentistry, Karaganda Medical University, Karaganda, Kazakhstan; 3 Departments of Otolaryngology and Ophthalmology, West Kazakhstan Marat Ospanov Medical University, Aktobe, Kazakhstan; 4 Pediatric Diseases Department, Astana Medical University, Astana, Kazakhstan; 5 Department of Natural Sciences, West Kazakhstan Marat Ospanov Medical University, Aktobe, Kazakhstan

**Keywords:** dental bleaching, desensitizing agents, photobiomodulation, potassium nitrate, tooth sensitivity, umbrella review

## Abstract

**Objective:**

This umbrella review aimed to synthesize and critically evaluate evidence from systematic reviews on interventions for the prevention and management of bleaching-induced tooth sensitivity, with emphasis on methodological quality, risk of bias, certainty of evidence, clinical relevance, and overlap of primary studies.

**Methods:**

An umbrella review was conducted following PRISMA 2020 guidance. PubMed, Scopus, Web of Science, the Cochrane Database of Systematic Reviews, Embase and LILACS Plus Collection were searched up to August 10, 2026. Methodological quality, risk of bias, certainty of evidence, clinical relevance, and overlap were assessed using AMSTAR-2, ROBIS, reported GRADE ratings, and the corrected covered area method.

**Results:**

Eighteen systematic reviews were included. Systemic analgesic and anti-inflammatory drugs consistently showed no clinically meaningful preventive effect. Potassium-nitrate-based interventions showed small reductions in sensitivity intensity, whereas effects on incidence were inconsistent. Evidence for fluoride was largely derived from combination formulations, limiting assessment of its independent effect. Calcium-based agents showed limited and low-certainty benefits. Photobiomodulation therapy (PBMT), historically referred to as low-level laser therapy (LLLT), appeared promising for reducing sensitivity intensity, but substantial protocol heterogeneity and variable risk of bias limited confidence in the findings. Overall, most interventions reduced sensitivity intensity rather than incidence.

**Conclusion:**

Bleaching-induced tooth sensitivity is common but usually transient. Systemic non-steroidal anti-inflammatory drugs appear ineffective. Potassium-nitrate-based interventions may provide modest relief in selected settings, whereas the independent benefit of fluoride remains uncertain because it was frequently evaluated as part of combination formulations. PBMT may also reduce sensitivity intensity, although protocol heterogeneity limits clinical recommendations. Standardized, high-quality randomized trials are needed.

**Systematic Review Registration:**

https://osf.io/9s8h7/.

## 1 Introduction

Peroxide-based tooth bleaching is a cornerstone of contemporary aesthetic dentistry, commonly performed using hydrogen peroxide or carbamide peroxide in both in-office and at-home protocols. Although generally safe and effective, bleaching-induced tooth sensitivity (TS) remains the most frequently reported adverse effect and represents a clinically relevant limitation of treatment (1, 2).

Recent evidence indicates that TS is common and influenced by bleaching protocols. A systematic review and meta-analysis comparing in-office and at-home bleaching reported lower TS intensity with at-home approaches, although differences in TS risk were not statistically significant and the certainty of evidence was low (3). Similarly, a network meta-analysis found that higher peroxide concentrations increased TS risk compared with lower-concentration regimens, although the overall quality of evidence remained limited (4).

Various pharmacological strategies have been proposed to reduce TS by modulating inflammation, nerve activity, or dentinal tubule permeability. These include non-steroidal anti-inflammatory drugs (NSAIDs) (5), potassium nitrate (1), fluoride-based agents, calcium-containing remineralizing products (6), bioactive glass (7) and multi-component desensitizers (8). Preoperative NSAIDs do not appear to significantly reduce TS incidence or intensity (2), while potassium nitrate shows small but statistically significant reductions in TS risk and intensity (1). Calcium-based desensitizers may reduce sensitivity intensity but have limited impact on TS risk, with low certainty of evidence (6).

Non-pharmacological interventions include photobiomodulation therapy (PBMT), historically referred to as low-level laser therapy (LLLT). PBMT may reduce post-bleaching pain symptoms without affecting whitening outcomes, but heterogeneity in wavelength, power, fluence, irradiation timing, treatment location, and application protocols limits direct clinical recommendations (9–12). Reviews of light activation or protocol optimization were considered contextual evidence and were not interpreted as active desensitizing interventions.

Despite the increasing number of systematic reviews addressing TS prevention, the available evidence remains heterogeneous and often limited by methodological concerns. In addition, many reviews include overlapping primary studies and employ different analytical approaches, outcome definitions, and methodological frameworks. As a result, interpretation of the overall evidence remains fragmented.

Therefore, this umbrella review aimed to synthesize evidence from systematic reviews and meta-analyses evaluating active pharmacological, topical, and light-based interventions for the prevention and management of bleaching-induced tooth sensitivity.

## 2 Methods

### 2.1 Protocol and registration

This umbrella review was designed and conducted in accordance with established methodological guidance for overviews of systematic reviews. The protocol was developed *a priori* to minimize bias in study selection, data extraction, and evidence synthesis.

The protocol for this umbrella review was prospectively registered in the Open Science Framework (OSF) on 23 February 2026 and was made available for peer-review verification. Any deviations from the protocol were documented and justified. Reporting followed the Preferred Reporting Items for Systematic Reviews and Meta-Analyses (PRISMA 2020) statement to ensure methodological transparency, reproducibility, and completeness of reporting.

### 2.2 Eligibility criteria

Eligibility criteria were predefined and applied at the level of systematic reviews. The criteria were structured according to the PICO framework (Population, Intervention, Comparator, Outcomes), with additional specifications regarding study design and publication characteristics. Vital bleaching was defined as external bleaching of vital teeth using in-office or at-home protocols. When reported, baseline tooth sensitivity, gingival recession, and other pre-existing conditions were extracted because they may modify the risk of bleaching-induced TS. Reviews focused only on dentin hypersensitivity unrelated to bleaching were excluded from efficacy conclusions (Table 1).

**Table 1:** PICO components and eligibility criteria.

Domain	Inclusion Criteria	Exclusion Criteria
Population (P)	Adults undergoing vital external dental bleaching (in-office or at-home) of vital teeth; baseline tooth sensitivity or pre-existing conditions (e.g., gingival recession) were extracted when reported.	Animal studies; *in vitro* studies; non-vital/internal bleaching; reviews in which post-bleaching TS could not be separated from dentin hypersensitivity.
Intervention (I)	Active pharmacological, topical, remineralizing, desensitizing, or light-based interventions intended to prevent or reduce bleaching-induced tooth sensitivity, including systemic analgesic and anti-inflammatory agents (e.g., NSAIDs and corticosteroids); potassium nitrate; fluoride-containing agents; calcium- and phosphate-based or bioactive remineralizing/desensitizing agents (e.g., CPP-ACP/ACP, hydroxyapatite or nano-hydroxyapatite, tricalcium phosphate, calcium sodium phosphosilicate/bioactive glass); arginine- and strontium-containing agents; other topical desensitizing formulations; and photobiomodulation therapy [PBMT, historically termed low-level laser therapy (LLLT)].	Reviews assessing only bleaching efficacy, whitening protocols, light activation, peroxide concentration, exposure time, or other protocol-optimization strategies without evaluating an active intervention for the prevention or management of bleaching-induced tooth sensitivity; interventions intended exclusively for dentin hypersensitivity unrelated to bleaching.
Comparator (C)	Placebo, sham treatment, no intervention, standard bleaching protocol, or alternative active intervention.	Reviews without a comparator group.
Outcomes (O)	Primary: Incidence (risk) of bleaching-induced tooth sensitivity (absolute risk, RR, OR); intensity of TS (VAS, NRS, validated scales). Secondary: Duration of TS; impact on bleaching efficacy (*Δ*E); adverse effects.	Reviews not reporting tooth sensitivity outcomes.
Study Design	Systematic reviews with or without meta-analysis; reviews primarily based on randomized controlled trials; explicit systematic methodology.	Narrative reviews; scoping reviews; rapid reviews; umbrella reviews; clinical guidelines; expert opinions; primary RCTs; non-systematic reviews.
Publication Type	Full-text articles published in peer-reviewed journals; any language.	Conference abstracts without full text; posters; theses/dissertations; non-peer-reviewed publications.

### 2.3 Electronic databases and search terms

Electronic searches were conducted in PubMed, Scopus, Web of Science Core Collection, the Cochrane Database of Systematic Reviews, Embase and LILACS Plus Collection from database inception to 10 August 2026. No language or publication-year restrictions were applied.

Search terms included combinations of keywords and controlled vocabulary related to tooth sensitivity, dental bleaching, pharmacological and desensitizing/remineralizing interventions (e.g., NSAIDs, analgesics, potassium nitrate, fluoride, CPP-ACP/ACP, hydroxyapatite, calcium sodium phosphosilicate/bioactive glass, arginine, strontium chloride, and other desensitizing or remineralizing agents), non-pharmacological interventions (e.g., laser therapy, PBMT, and LLLT), and study design (systematic review and meta-analysis). MeSH, Emtree, and database-specific indexing terms were used when available, and Boolean operators (“AND”, “OR”) were used to combine terms (Table 2).

**Table 2:** Search strategy used in electronic databases 10/08/2026.

Database	Search String/Query	Filters Applied and Search Results
PubMed	(“Tooth Sensitivity"[Mesh] OR “Dentin Sensitivity"[Mesh] OR “tooth sensitivity"[Title/Abstract] OR “dentin sensitivity"[Title/Abstract] OR “tooth hypersensitivity"[Title/Abstract]) AND (“Tooth Bleaching"[Mesh] OR “Dental Bleaching"[Title/Abstract] OR “Tooth Whitening"[Title/Abstract] OR “Teeth Whitening"[Title/Abstract] OR bleaching[Title/Abstract] OR whitening[Title/Abstract]) AND (“Anti-Inflammatory Agents, Non-Steroidal"[Mesh] OR “Ibuprofen"[Mesh] OR ibuprofen[Title/Abstract] OR “Naproxen"[Mesh] OR naproxen[Title/Abstract] OR “Diclofenac"[Mesh] OR diclofenac[Title/Abstract] OR “Ketoprofen"[Mesh] OR ketoprofen[Title/Abstract] OR “Analgesics"[Mesh] OR analgesics[Title/Abstract] OR “Potassium Nitrate"[Mesh] OR “potassium nitrate"[Title/Abstract] OR “Fluorides"[Mesh] OR fluoride[Title/Abstract] OR “sodium fluoride"[Title/Abstract] OR desensitizing[Title/Abstract] OR desensitising[Title/Abstract] OR “desensitizing agent"[Title/Abstract] OR “desensitizing agents"[Title/Abstract] OR “desensitising agent"[Title/Abstract] OR “desensitising agents"[Title/Abstract] OR remineralizing[Title/Abstract] OR remineralising[Title/Abstract] OR “remineralizing agent"[Title/Abstract] OR “remineralizing agents"[Title/Abstract] OR “remineralising agent"[Title/Abstract] OR “remineralising agents"[Title/Abstract] OR “CPP-ACP"[Title/Abstract] OR “casein phosphopeptide amorphous calcium phosphate"[Title/Abstract] OR “casein phosphopeptide-amorphous calcium phosphate"[Title/Abstract] OR “amorphous calcium phosphate"[Title/Abstract] OR hydroxyapatite[Title/Abstract] OR “nano-hydroxyapatite"[Title/Abstract] OR nanohydroxyapatite[Title/Abstract] OR “calcium sodium phosphosilicate"[Title/Abstract] OR bioglass[Title/Abstract] OR “bioactive glass"[Title/Abstract] OR “tricalcium phosphate"[Title/Abstract] OR arginine[Title/Abstract] OR “strontium chloride"[Title/Abstract] OR laser[Title/Abstract] OR photobiomodulation[Title/Abstract] OR PBMT[Title/Abstract] OR “low-level laser therapy"[Title/Abstract] OR LLLT[Title/Abstract]) AND (“Systematic Review"[Publication Type] OR “Meta-Analysis"[Publication Type] OR “systematic review"[Title/Abstract] OR “meta-analysis"[Title/Abstract])	Filters applied: None (no restrictions on publication year or language). Search results: 22 records
Scopus	TITLE-ABS-KEY(“tooth sensitivity” OR “dentin sensitivity” OR “tooth hypersensitivity”) AND TITLE-ABS-KEY(“tooth bleaching” OR “dental bleaching” OR “tooth whitening” OR “teeth whitening” OR bleaching OR whitening) AND TITLE-ABS-KEY(NSAIDs OR ibuprofen OR diclofenac OR ketoprofen OR naproxen OR analgesics OR “potassium nitrate” OR fluoride OR “sodium fluoride” OR desensitizing OR desensitising OR “desensitizing agent” OR “desensitizing agents” OR “desensitising agent” OR “desensitising agents” OR remineralizing OR remineralising OR “remineralizing agent” OR “remineralizing agents” OR “remineralising agent” OR “remineralising agents” OR “CPP-ACP” OR “casein phosphopeptide amorphous calcium phosphate” OR “casein phosphopeptide-amorphous calcium phosphate” OR “amorphous calcium phosphate” OR hydroxyapatite OR “nano-hydroxyapatite” OR nanohydroxyapatite OR “calcium sodium phosphosilicate” OR bioglass OR “bioactive glass” OR “tricalcium phosphate” OR arginine OR “strontium chloride” OR laser OR photobiomodulation OR PBMT OR “low-level laser therapy” OR LLLT) AND TITLE-ABS-KEY(“systematic review” OR “meta-analysis”)	Filters applied: Document type: Article and Review; No language or year restrictions. Search results: 28 records
Web of Science Core Collection	TS = (“tooth sensitivity” OR “dentin sensitivity” OR “tooth hypersensitivity”) AND TS = (“tooth bleaching” OR “dental bleaching” OR “tooth whitening” OR “teeth whitening” OR bleaching OR whitening) AND TS = (NSAIDs OR ibuprofen OR diclofenac OR ketoprofen OR naproxen OR analgesics OR “potassium nitrate” OR fluoride OR “sodium fluoride” OR desensitizing OR desensitising OR “desensitizing agent” OR “desensitizing agents” OR “desensitising agent” OR “desensitising agents” OR remineralizing OR remineralising OR “remineralizing agent” OR “remineralizing agents” OR “remineralising agent” OR “remineralising agents” OR “CPP-ACP” OR “casein phosphopeptide amorphous calcium phosphate” OR “casein phosphopeptide-amorphous calcium phosphate” OR “amorphous calcium phosphate” OR hydroxyapatite OR “nano-hydroxyapatite” OR nanohydroxyapatite OR “calcium sodium phosphosilicate” OR bioglass OR “bioactive glass” OR “tricalcium phosphate” OR arginine OR “strontium chloride” OR laser OR photobiomodulation OR PBMT OR “low-level laser therapy” OR LLLT) AND TS = (“systematic review” OR “meta-analysis”)	Filters applied: Document type: Article and Review; No language or year restrictions. Search results: 35 records
Cochrane Database of Systematic Reviews	(“tooth sensitivity” OR “dentin sensitivity” OR “tooth hypersensitivity”) AND (“tooth bleaching” OR “dental bleaching” OR “tooth whitening” OR “teeth whitening” OR bleaching OR whitening) AND (NSAIDs OR ibuprofen OR diclofenac OR ketoprofen OR naproxen OR analgesics OR “potassium nitrate” OR fluoride OR “sodium fluoride” OR desensitizing OR desensitising OR “desensitizing agent” OR “desensitizing agents” OR remineralizing OR remineralising OR “remineralizing agent” OR “remineralizing agents” OR “CPP-ACP” OR “casein phosphopeptide amorphous calcium phosphate” OR “casein phosphopeptide-amorphous calcium phosphate” OR “amorphous calcium phosphate” OR hydroxyapatite OR “nano-hydroxyapatite” OR nanohydroxyapatite OR “calcium sodium phosphosilicate” OR bioglass OR “bioactive glass” OR “tricalcium phosphate” OR arginine OR “strontium chloride” OR laser OR photobiomodulation OR PBMT OR “low-level laser therapy” OR LLLT)	Filters applied: Cochrane Reviews; No language or year restrictions. Search results:4 records
Embase (via Elsevier)	(“tooth sensitivity”/exp OR ‘dentin sensitivity’/exp OR ‘tooth hypersensitivity’:ti,ab OR ‘dentin sensitivity’:ti,ab OR ‘tooth sensitivity’:ti,ab) AND (‘tooth bleaching’/exp OR ‘dental bleaching’:ti,ab OR ‘tooth whitening’:ti,ab OR ‘teeth whitening’:ti,ab OR bleaching:ti,ab OR whitening:ti,ab) AND (‘nonsteroidal antiinflammatory agent’/exp OR nsaids:ti,ab OR ibuprofen:ti,ab OR diclofenac:ti,ab OR ketoprofen:ti,ab OR naproxen:ti,ab OR analgesics:ti,ab OR ‘potassium nitrate’:ti,ab OR fluoride:ti,ab OR ’sodium fluoride’:ti,ab OR desensitizing:ti,ab OR desensitising:ti,ab OR ‘desensitizing agent’:ti,ab OR ‘desensitizing agents’:ti,ab OR ‘desensitising agent’:ti,ab OR ‘desensitising agents':ti,ab OR remineralizing:ti,ab OR remineralising:ti,ab OR ‘remineralizing agent’:ti,ab OR ‘remineralizing agents':ti,ab OR ‘CPP-ACP’:ti,ab OR ‘casein phosphopeptide amorphous calcium phosphate’:ti,ab OR ‘casein phosphopeptide-amorphous calcium phosphate’:ti,ab OR ‘amorphous calcium phosphate’:ti,ab OR hydroxyapatite:ti,ab OR ‘nano-hydroxyapatite’:ti,ab OR nanohydroxyapatite:ti,ab OR ‘calcium sodium phosphosilicate’:ti,ab OR bioglass:ti,ab OR ‘bioactive glass':ti,ab OR ‘tricalcium phosphate’:ti,ab OR arginine:ti,ab OR ‘strontium chloride’:ti,ab OR laser:ti,ab OR photobiomodulation:ti,ab OR pbmt:ti,ab OR ‘low-level laser therapy’:ti,ab OR lllt:ti,ab) AND (‘systematic review’/de OR ‘meta analysis'/de OR ‘systematic review’:ti,ab OR ‘meta-analysis':ti,ab)	Filters applied: Review; Search results: 10 records
LILACS Plus Collection (supplementary regional search)	(“tooth sensitivity” OR “dentin sensitivity” OR “tooth hypersensitivity” OR “sensibilidade dentária”) AND (“tooth bleaching” OR “dental bleaching” OR “tooth whitening” OR bleaching OR clareamento) AND (NSAIDs OR ibuprofen OR naproxen OR diclofenac OR ketoprofen OR analgesics OR “potassium nitrate” OR fluoride OR “sodium fluoride” OR desensitizing OR desensitising OR dessensibilizante OR remineralizing OR remineralising OR remineralização OR “CPP-ACP” OR “casein phosphopeptide amorphous calcium phosphate” OR “amorphous calcium phosphate” OR hydroxyapatite OR hidroxiapatita OR “nano-hydroxyapatite” OR nanohydroxyapatite OR “calcium sodium phosphosilicate” OR bioglass OR “bioactive glass” OR “vidro bioativo” OR “tricalcium phosphate” OR arginine OR arginina OR “strontium chloride” OR laser OR photobiomodulation OR PBMT OR “low-level laser therapy” OR LLLT) AND (“systematic review” OR “meta-analysis” OR “revisão sistemática” OR metanálise)	No language or year restrictions. Supplementary regional search performed to improve coverage of Latin American literature. Search results: 14 records.

### 2.4 Study selection process

The study selection process is illustrated in Figure 1. Records retrieved from the electronic databases were imported into reference management software, and duplicate records were removed before screening. Titles and abstracts were screened independently by two reviewers according to the predefined eligibility criteria.

**Figure 1:**
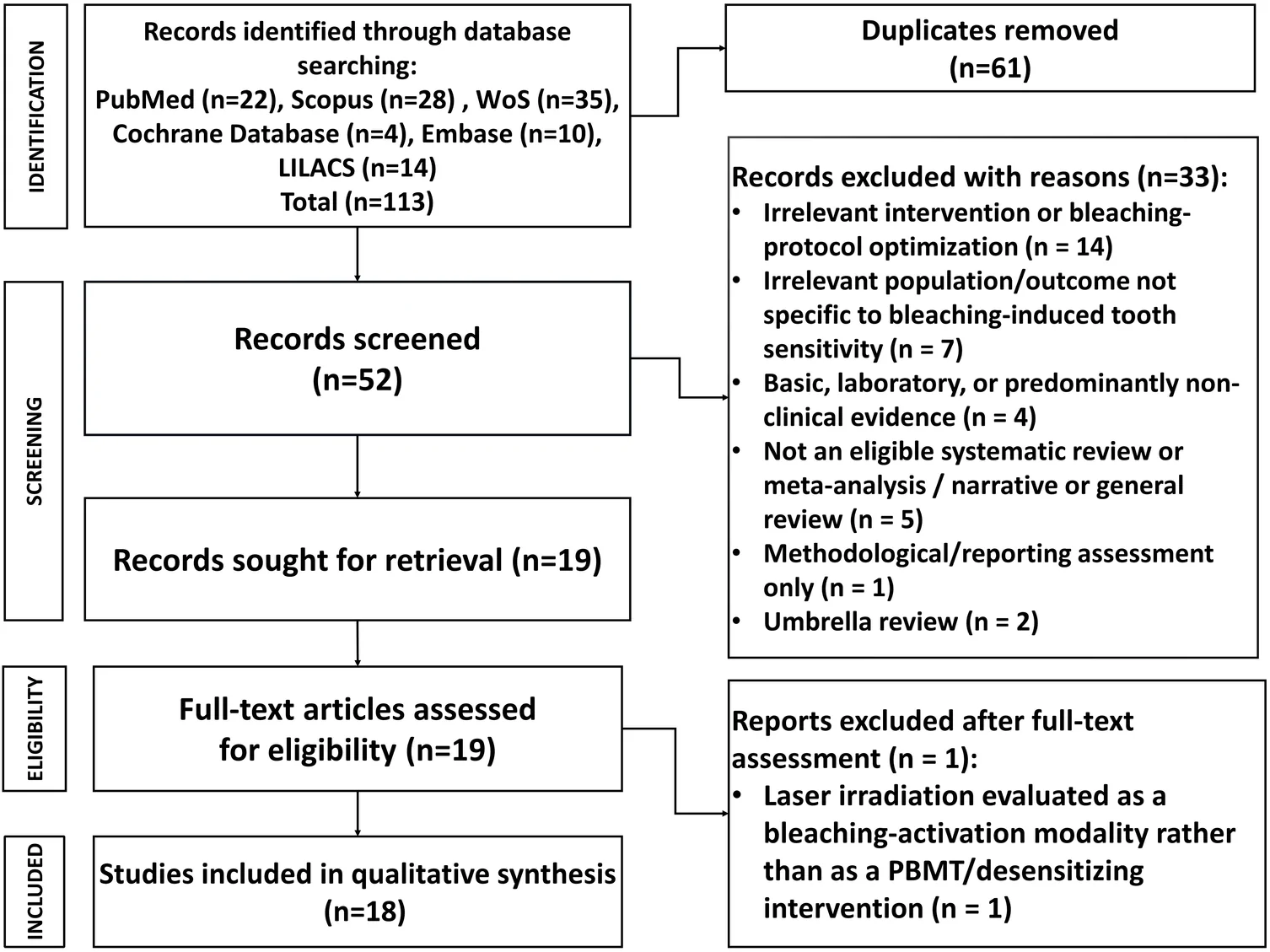
PRISMA 2020 flow diagram of the study selection process.

Potentially eligible articles were retrieved and assessed in full text. Full-text screening was performed independently by two reviewers, and reasons for exclusion were recorded. Disagreements at any stage of the selection process were resolved through discussion and consensus, with consultation from a third reviewer when necessary. Inter-reviewer agreement for study selection was excellent (Cohen's *κ* = 0.87). Only systematic reviews and meta-analyses that fulfilled all inclusion criteria and did not meet any exclusion criteria were included in the final qualitative synthesis.

### 2.5 Data extraction

Data extraction was independently performed by two reviewers using a standardized form. Extracted data included study characteristics, population details, bleaching type (in-office or at-home), peroxide concentration, tray type when available, interventions, comparators, outcome scales, effect estimates, confidence intervals, I^2^ values, GRADE ratings, ROBIS judgments, and methodological quality indicators. For systematic reviews evaluating PBMT, additional protocol-level data were extracted where available, including wavelength, output power, energy per point, fluence, irradiation time, number and location of irradiation points, timing relative to bleaching, and number of treatment applications or sessions. Parameters not reported by the source systematic reviews were recorded as NR and were not inferred from the primary studies.

### 2.6 Assessment of methodological quality

The methodological quality of the included systematic reviews and meta-analyses was assessed using AMSTAR-2 (A Measurement Tool to Assess Systematic Reviews 2), a validated instrument designed for evaluating systematic reviews of healthcare interventions. AMSTAR-2 includes 16 domains, including seven critical domains related to protocol registration, adequacy of the literature search, justification of excluded studies, assessment of risk of bias, appropriateness of meta-analytical methods, and evaluation of publication bias.

In addition, the risk of bias of the included systematic reviews was assessed using the ROBIS (Risk Of Bias In Systematic Reviews) tool. ROBIS evaluates potential bias across four domains: (1) study eligibility criteria, (2) identification and selection of studies, (3) data collection and study appraisal, and (4) synthesis and findings, followed by an overall judgment of risk of bias.

Two reviewers independently performed the AMSTAR-2 and ROBIS assessments according to the published guidance for each tool. For AMSTAR-2, domains were rated as ‘Yes,’ ‘Partial Yes,’ or ‘No.’ For ROBIS, risk-of-bias judgments were categorized as ‘Low,’ ‘High,’ or ‘Unclear.’ Inter-reviewer agreement was calculated before consensus for quality appraisal (AMSTAR-2 *κ*=0.82; ROBIS *κ*=0.79). Any disagreements were resolved through discussion and consensus, with consultation from a third reviewer when necessary.

### 2.7 Assessment of overlap across reviews

To evaluate the degree of overlap among primary studies included in the systematic reviews and meta-analyses, a citation matrix was constructed, in which rows represented primary studies and columns represented the included systematic reviews and meta-analyses. Each cell indicated whether a given primary study was included in a specific review.

The extent of overlap was quantified using the Corrected Covered Area (CCA) method, as described by Pieper et al. (2014) (13). The CCA provides a standardized measure of redundancy across reviews, calculated using the formula:

CCA=[(N−r)/(r×c−r)]×100%

where *N* represents the total number of included primary study occurrences across reviews, *r* represents the number of unique primary studies, and *c* represents the number of systematic reviews and meta-analyses. The degree of overlap was interpreted according to established thresholds: 0%–5% (slight overlap), 5%–10% (moderate overlap), 10%–15% (high overlap), and >15% (very high overlap).

In addition to the overall CCA, intervention-specific CCA values were calculated for clinical intervention categories represented by at least two systematic reviews addressing the same intervention category. These analyses were performed for systemic NSAIDs/analgesics, PBMT/LLLT, and potassium nitrate/fluoride-based interventions. Intervention categories represented by a single dedicated review were not assigned a CCA because overlap cannot be quantified when *c* *=* *1*.

### 2.8 Data synthesis and analysis

Extracted data included authorship, publication year, number and type of primary studies, interventions, comparators, outcomes, and main findings, which were summarized in structured tables and narrative form.

For reviews reporting meta-analyses, pooled effect estimates were interpreted according to their direction, magnitude, 95% confidence intervals, and reported heterogeneity I^2^. Statistical significance was considered together with clinical relevance. Because the included reviews did not provide a validated minimal clinically important difference (MCID) specific to bleaching-induced tooth sensitivity, the approximately 1-point threshold on a 10-point visual analog scale (VAS), derived from broader acute-pain literature, was treated only as an extrapolated clinical benchmark. Accordingly, effects of approximately 1 VAS point or less were described as small absolute effects, and their clinical perceptibility was considered uncertain rather than being classified as below a condition-specific MCID.

When available, GRADE certainty ratings were extracted as reported by the original systematic reviews. No *de novo* GRADE assessment was performed at the umbrella-review level. For interventions assessed by multiple reviews, an umbrella-level interpretation of evidence certainty was based on methodological quality (AMSTAR-2), risk of bias (ROBIS), consistency of findings, precision of estimates, clinical meaningfulness, reported heterogeneity I^2^, and degree of overlap between reviews. A qualitative quality-based sensitivity interpretation was performed to examine whether methodological limitations of the source reviews materially affected intervention-level conclusions. No reviews were excluded or numerically weighted, and no scoring-based adjustment was applied. All eligible reviews remained in the synthesis. For each intervention, findings from reviews with fewer methodological concerns were qualitatively compared with the complete evidence base. Greater interpretive weight was given to reviews with lower ROBIS concern, fewer limitations in critical AMSTAR-2 domains, appropriate assessment of publication bias where applicable, adequately addressed heterogeneity, and higher GRADE certainty when GRADE had been reported by the source review. The comparison was used solely to determine whether consideration of review quality altered the direction, magnitude, or confidence of the intervention-level conclusion and was not treated as a formal statistical sensitivity analysis.

Reference management was performed using Zotero (version 8.0.3; Corporation for Digital Scholarship, Vienna, VA, USA). Data management and overlap analyses were conducted using R software (version 4.4.1; R Foundation for Statistical Computing, Vienna, Austria) within RStudio (Posit Software, Boston, MA, USA), including construction of citation matrices and graphical visualizations.

## 3 Results

### 3.1 Study selection

The database searches identified 113 records: PubMed (*n* = 22), Scopus (*n* = 28), Web of Science Core Collection (*n* = 35), the Cochrane Database of Systematic Reviews (*n* = 4), Embase (*n* = 10), and LILACS (*n* = 14). After removal of 61 duplicate records, 52 records remained for title and abstract screening. Thirty-three records were excluded at this stage, and 19 reports were assessed for eligibility in full text. One report was excluded. Consequently, 18 systematic reviews and meta-analyses fulfilled the eligibility criteria and were included in the umbrella review. The study selection process is presented in Figure 1 according to PRISMA 2020.

### 3.2 Characteristics of included systematic reviews and meta-analyses

#### 3.2.1 Pharmacological interventions

Several systematic reviews evaluated systemic anti-inflammatory and analgesic agents administered before or during dental bleaching procedures. Across these reviews, systemic NSAIDs and corticosteroids consistently showed no clinically meaningful reduction in the risk or intensity of bleaching-induced TS and did not affect bleaching efficacy (2, 5, 14–17). This conclusion remained stable in the quality-based sensitivity interpretation because the direction of effect was consistent even among reviews with stronger methodology. The detailed characteristics of the included systematic reviews and meta-analyses are presented in Table 3.

**Table 3:** Characteristics of included systematic reviews and meta-analyses.

Author (Year)	No. of studies	Population/sample size	Intervention	Comparator	Outcomes	Principal pooled effect estimate (95% CI)	I^2^	Main findings	Quality assessment
Almassri et al. (2019) (15)	7 RCTs	Adults undergoing in-office bleaching; *n* = 324	Oral anti-inflammatory drugs: NSAIDs and corticosteroids	Placebo	TS risk/intensity; color change	TS risk: RR = 0.98 (95% CI, 0.87–1.10); TS intensity during bleaching: SMD=0.16 (95% CI, −0.13 to 0.45)	8%; 0%, respectively	No significant reduction in TS risk or intensity; no effect on bleaching efficacy	Cochrane RoB: 6 studies high quality, 1 study low quality; ROBIS unclear; GRADE NR
Cabral et al. (2024) (21)	5 RCTs	In-office or at-home bleaching; *n* = 387	Desensitizing toothpastes: KNO₃, strontium chloride, calcium-based agents, arginine	Regular/placebo toothpaste or no desensitizer	TS mainly by VAS/NRS	Not pooled	NR	Effectiveness depended on bleaching protocol; benefit seen in some home and single-session in-office protocols	Cochrane RoB: low to moderate; ROBIS low; GRADE NR
Carneiro et al. (2022) (10)	6 RCTs	Adults undergoing in-office bleaching; *n* = 6–66 per study	PBMT/LLLT using red or infrared diode lasers	Placebo laser, bleaching alone, or alternative treatment	TS by VAS, modified VAS, or VRS	Not pooled	I^2^ = 87%^a^	Most studies showed reduced post-bleaching TS, but evidence was heterogeneous	Cochrane RoB: mostly low, 1 study unclear, 1 study high; ROBIS low; GRADE NR
Carregosa Santana et al. (2019) (16)	11 RCTs	Adults undergoing in-office bleaching; *n* = 712	Oral analgesic/anti-inflammatory drugs	Placebo	TS risk and intensity	TS risk: RR = 0.97 (95% CI, 0.93–1.03); TS intensity ≤1 h (VAS, low-risk subgroup): MD = −0.33 (95% CI, −0.85 to 0.19); TS intensity 1–24 h (VAS): MD = −0.35 (95% CI, −0.77 to 0.08)	0%; 36%; 0%, respectively	No significant or clinically relevant reduction in TS risk or intensity	OHAT RoB: 9 low, 2 high; ROBIS NR; GRADE high
Costa et al. (2020) (17)	7 RCTs	In-office bleaching; *n* = 359	Preemptive oral analgesics/NSAIDs/corticosteroids	Placebo	TS risk and intensity	TS incidence: RR = 1.03 (95% CI, 0.92–1.15); TS intensity immediately after bleaching: MD = −0.90 (95% CI, −1.63 to −0.16); TS intensity 1–24 h: MD = 0.05 (95% CI, −0.61 to 0.72)	21%; 67%; 0%, respectively	No meaningful reduction in bleaching-induced TS; small immediate effect had limited relevance	Cochrane RoB mostly low; ROBIS NR; GRADE NR
Costa et al. (2025) (5)	16 RCTs	In-office bleaching; *n* = 823	Systemic analgesic/anti-inflammatory drugs	Placebo	Pain/TS scores, TS incidence, color outcomes	VAS: MD = −0.00 (95% CI, −0.01 to 0.00); VAS at 1 h: MD = −0.49 (95% CI, −0.83 to −0.16); TS incidence: RR = 0.99 (95% CI, 0.91–1.07)	81%; 0%; 0%, respectively	Minimal, clinically insignificant benefit; possible slight negative effect on bleaching efficacy	RoB-2: low; ROBIS low; GRADE moderate–high
Costacurta et al. (2020) (20)	6 trials	At-home CP bleaching; *n* = 133–211 depending on outcome	CP gels containing potassium nitrate	CP gels without potassium nitrate	TS risk/intensity; color change	TS risk: RR = 0.93 (95% CI, 0.73–1.19); TS intensity: SMD=−0.10 (95% CI, −0.36 to 0.16)	0%; 0%, respectively	No significant reduction in TS or effect on bleaching efficacy	Cochrane RoB: mostly unclear; ROBIS NR; GRADE moderate–low
da Silva et al. (2022) (2)	7 RCTs	In-office bleaching; ≈270 participants	Preoperative oral NSAIDs	Placebo	TS intensity and incidence	During bleaching: MD = −0.30 (95% CI, −0.88 to 0.29); immediately after: MD = −0.29 (95% CI, −0.75 to 0.17); up to 1 h: MD = −0.94 (95% CI, −2.06 to 0.18); 1–24 h: MD = −0.11 (95% CI, −0.90 to 0.68); TS incidence: OR = 1.15 (95% CI, 0.69–1.92)	3%; 73%; 0%; 0%; 0%, respectively	NSAIDs did not reduce TS during or after bleaching	Cochrane RoB mostly low; ROBIS NR; GRADE NR
de Barros Silva et al. (2022) (9)	5 RCTs	In-office bleaching; *n* = 288	PBMT	Placebo/sham laser	TS and color change	After 1st bleaching session: Cohen's d = −0.32 (95% CI, −0.46 to −0.18); after 2nd session: d = −0.30 (95% CI, −0.46 to −0.15); after 3rd session: d = −0.82 (95% CI, −1.06 to −0.58)	80%; 69%; 94%, respectively	PBMT reduced TS after bleaching sessions without affecting color change	JBI RoB mostly low; ROBIS low; GRADE moderate–low
de Oliveira et al. (2024) (22)	4 RCTs	In-office bleaching; *n* = 258/516 hemi-arches	Topical drugs: Otosporin, ibuprofen + arginine, dipyrone, eugenol	Placebo/control on opposite hemi-arch	TS by VAS/NRS; color change	TS ≤1 h: MD = −0.39 (95% CI, −0.89 to 0.11); TS at 24 h: MD = −0.26 (95% CI, −0.71 to 0.18); TS at 48 h: MD = 0.00 (95% CI, −0.16 to 0.16)	10%; 0%; 1%, respectively	Topical drugs did not reduce TS and did not impair color change	RoB-2: low; ROBIS unclear; GRADE moderate/high
Faria-e-Silva et al. (2015) (14)	3 RCTs	In-office bleaching; *n* = 91	Preventive oral NSAIDs	Placebo	TS risk and intensity	TS intensity ≤1 h: MD = −8.80 (95% CI, −23.35 to 5.75); TS intensity 1–24 h: MD = −4.13 (95% CI, −16.24 to 7.97); TS risk: RR = 1.05 (95% CI, 0.82–1.34)	33%; 0%; 0%, respectively	NSAIDs did not reduce TS risk or intensity; evidence limited	Cochrane RoB; ROBIS NR; GRADE low
Favoreto et al. (2023) (6)	22 RCTs	Adults undergoing in-office or at-home bleaching; *n* = 652–703	Calcium-containing bioactive desensitizers	Placebo/no desensitizer	TS risk/intensity; color change	TS risk: RR = 0.95 (95% CI, 0.90–1.01); TS intensity: MD = −0.98 VAS units (95% CI, −1.36 to −0.60)	27%; 48%, respectively	No TS risk reduction; small TS intensity reduction; no color effect	RoB-2: mostly high; ROBIS low; GRADE low–very low
Giannakopoulou et al. (2024) (11)	6 RCTs	In-office bleaching; *n* = 308	PBMT using diode lasers	Placebo, no treatment, or desensitizers	TS by VAS/VRS/NRS	Not pooled	NR	Most studies favored PBMT, but heterogeneity prevented meta-analysis	RoB-2: some concerns/high; ROBIS low; GRADE NR
Krishnakumar et al. (2022) (8)	13 RCTs	In-office bleaching; *n* = 841	Desensitizing agents before bleaching, including potassium nitrate ± sodium fluoride, calcium-based agents, glutaraldehyde-containing agents, desensitizing dentifrices	Placebo/no treatment	TS and color change	Not pooled	NR	Desensitizers reduced immediate TS; no clear effect on color; evidence heterogeneous	Cochrane RoB; ROBIS high; GRADE NR
Martini et al. (2021) (1)	24 RCTs	At-home or in-office bleaching; adult patients	Topical potassium nitrate agents	Placebo/control	TS risk/intensity; color change	TS risk: RR = 0.88 (95% CI, 0.78–0.98); TS intensity (VAS): MD = −0.77 (95% CI, −1.34 to −0.19); TS intensity: MD = −0.36 (95% CI, −0.61 to −0.12)	59%; 57%; 66%, respectively	Small reduction in TS risk and intensity; no effect on bleaching efficacy	Cochrane RoB; ROBIS low; GRADE moderate–low
Rezende et al. (2019) (19)	8 RCTs	At-home bleaching; *n* = 211	Bleaching gels with incorporated desensitizers	Bleaching gels without desensitizers	TS, color, gingival irritation	TS risk: RR = 0.99 (95% CI, 0.74–1.33); TS intensity: SMD=−0.04 (95% CI, −0.79 to 0.70)	0%; 81%, respectively	No significant benefit for TS, color change, or gingival irritation	Cochrane RoB: unclear; ROBIS low; GRADE moderate–very low
Siqueira et al. (2024) (12)	10 RCTs	Adults undergoing in-office bleaching; *n* = 409	LLLT/PBMT, alone or combined with other desensitizing agents, including sodium fluoride, potassium nitrate, and strontium chloride	Placebo, no intervention, or comparator desensitizing treatment depending on study	Post-bleaching TS measured immediately, at 24 h, 48 h, and up to 7 days using VAS, modified VAS, or VRS	Not pooled	NR	Most included studies reported reduced post-bleaching TS with LLLT/PBMT, particularly during the first few days; however, interpretation was limited by heterogeneity in laser parameters, bleaching protocols, outcome scales, and concomitant desensitizing agents.	Cochrane RoB: NR; GRADE NR
Wang et al. (2015) (18)	10 trials	At-home or in-office bleaching; *n* = 394	Potassium nitrate and/or sodium fluoride	Placebo/no desensitizer	TS incidence/intensity; color change	TS incidence: OR = 0.45 (95% CI, 0.28–0.73); TS intensity: SMD=−0.47 (95% CI, −0.77 to −0.18)	16%; 41%, respectively	Reduced TS risk and intensity without compromising bleaching efficacy	Cochrane RoB: 1 study low risk, 7 studies unclear risk, 2 studies high risk; ROBIS NR; GRADE NR

CI, confidence interval; CP, carbamide peroxide; GRADE, Grading of Recommendations Assessment, Development and Evaluation; HP, hydrogen peroxide; JBI, Joanna Briggs Institute; KNO₃, potassium nitrate; MD, mean difference; NaF, sodium fluoride; NR, not reported; NSAIDs, non-steroidal anti-inflammatory drugs; OHAT, Office of Health Assessment and Translation; PBM, photobiomodulation; RCT, randomized controlled trial; RoB, risk of bias; ROBIS, Risk of Bias in Systematic Reviews; RR, relative risk; TS, tooth sensitivity; VAS, visual analog scale.

a I^2^ = 87% was reported by the source review as evidence of substantial heterogeneity contributing to the inability to perform a valid meta-analysis; no pooled effect estimate was ultimately reported.

Detailed PBMT irradiation parameters extracted from the source systematic reviews are presented in Supplementary Table S2.

Topical desensitizing agents have been more frequently investigated. Reviews evaluating potassium-nitrate-based interventions reported modest reductions in TS intensity, although effects on incidence varied across bleaching setting, peroxide concentration, delivery method, and timing of application. Fluoride was frequently evaluated in combination with potassium nitrate or other desensitizing agents; therefore, its independent contribution to the observed effects could not be reliably determined (1, 8, 18–21). The effect was generally clearer for intensity than for incidence, and no consistent impairment of bleaching efficacy was reported.

Other desensitizing agents have also been assessed. Calcium-containing bioactive agents were interpreted by compound class rather than as a single mechanism: CPP-ACP/ACP formulations, hydroxyapatite, tricalcium phosphate, and calcium sodium phosphosilicate/bioglass showed, at most, small reductions in sensitivity intensity without consistent effects on TS incidence, with the source review reporting low to very low certainty due to heterogeneity and risk of bias (6). Evidence from silver diamine fluoride reviews was not used for bleaching-specific efficacy conclusions because those reviews primarily addressed dentin hypersensitivity rather than peroxide-mediated post-bleaching TS.

#### 3.2.2 Non-pharmacological interventions

Non-pharmacological strategies primarily involved PBMT. Across the PBMT-focused systematic reviews, substantial heterogeneity was observed in treatment parameters. Reported wavelengths ranged approximately from 660 to 900 nm, while laser output power varied from 40 to 500 mW across the available protocols. Fluence also varied considerably, and some studies used hybrid LED/infrared laser systems rather than a single PBMT source. PBMT was administered before bleaching, after bleaching, during bleaching, or both before and after bleaching, and irradiation sites included cervical, coronal, apical, and periapical regions depending on the protocol. Energy per point, irradiation time, number and location of irradiation points, and the number of PBMT applications were not consistently reported across all source reviews (9–12). No reproducible optimal combination of wavelength, power, dose, timing, irradiation site, or treatment schedule could therefore be identified. Detailed protocol characteristics are presented in Supplementary Table S2.

### 3.3 Methodological quality of included reviews

The methodological quality of the included systematic reviews and meta-analyses was assessed using AMSTAR-2 (Figure 2), and risk of bias was assessed using ROBIS (Figure 3). Overall, considerable variability in methodological rigor was observed across the 18 included reviews. AMSTAR-2 was interpreted as a measure of methodological quality, whereas ROBIS was interpreted separately as risk of bias in the review process and conclusions.

**Figure 2:**
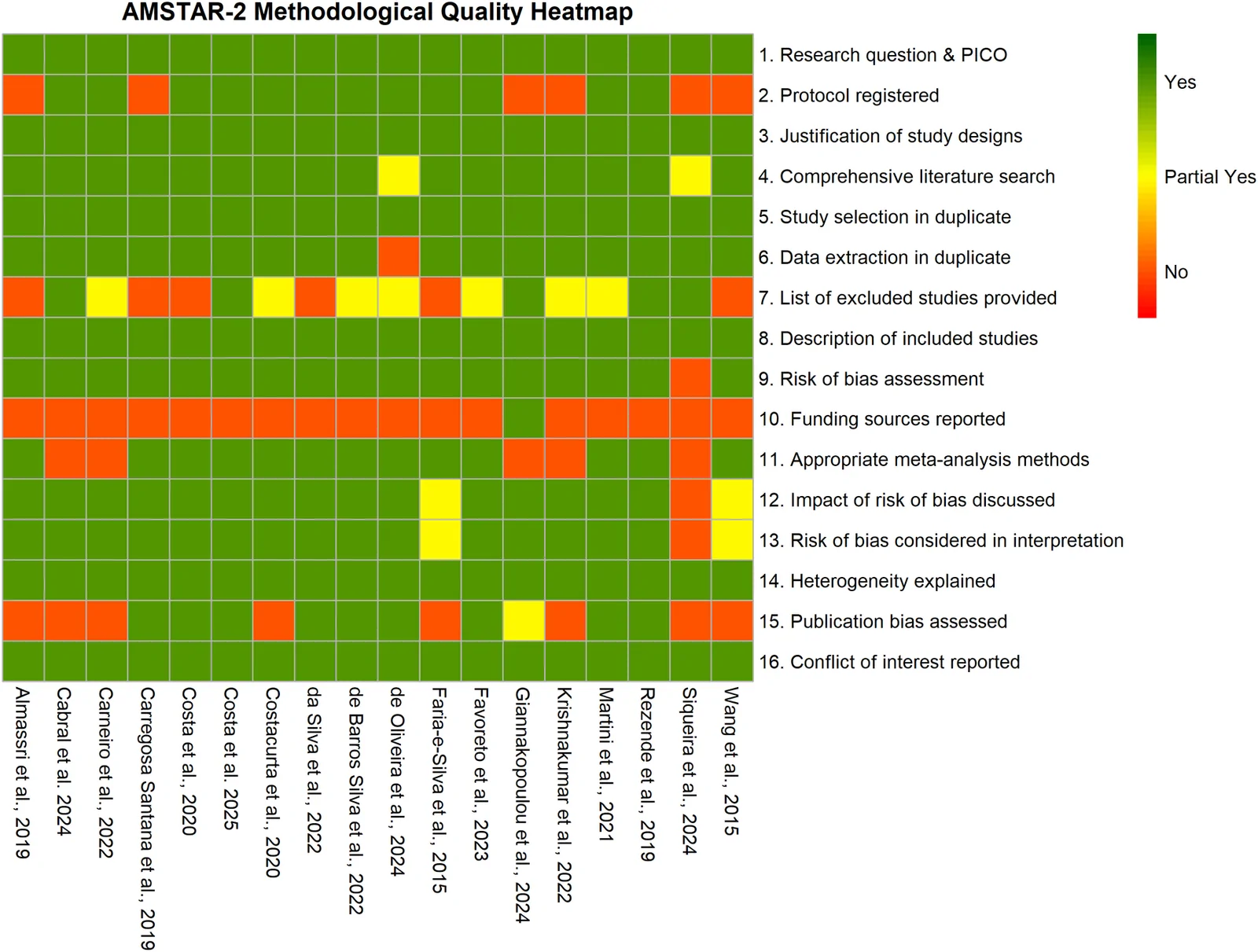
AMSTAR-2 methodological quality assessment heatmap of included systematic reviews.

**Figure 3:**
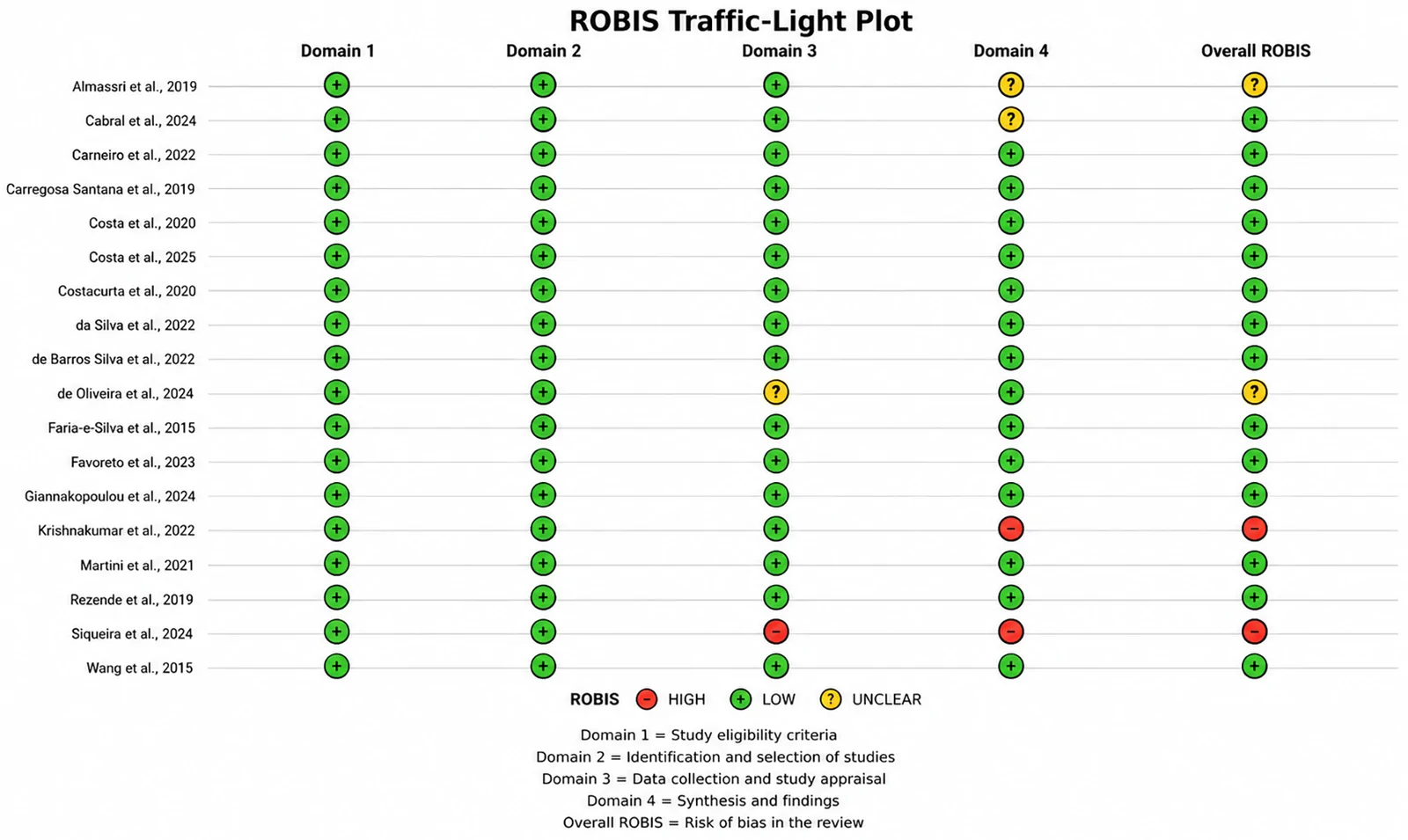
ROBIS traffic-light plot showing risk-of-bias judgments across the included systematic reviews.

Reviews assessing topical and other desensitizing agents showed methodological variability. Although several reviews applied risk of bias tools and quantitative synthesis, heterogeneity across interventions and variability in outcome measurement limited interpretability (1, 8, 18–22). Reviews evaluating calcium-based desensitizers reported substantial risk of bias in the included trials and low or very low certainty for several outcomes because of inconsistency and imprecision (6). Similarly, some reviews reported moderate certainty of evidence but did not consistently integrate risk of bias assessments into interpretation of findings (18–20).

Reviews evaluating photobiomodulation and laser-based therapies also demonstrated methodological limitations, including small numbers of included trials, heterogeneity across protocols, and incomplete reporting of publication bias assessment (9–12).

Across all included reviews, the most frequently unmet AMSTAR-2 critical domains were absence of protocol registration, inadequate reporting of excluded studies, limited consideration of risk of bias in interpretation of results, and incomplete assessment of publication bias. Reporting of funding sources for the included primary studies was also particularly limited. Only Giannakopoulou et al. (11) reported funding information for the primary studies, whereas the remaining reviews did not provide this information. Overall ROBIS judgments were low for 14 of 18 reviews (77.8%), unclear for two (11.1%), and high for two (11.1%). High overall risk of bias was confined to reviews with important limitations in study appraisal, synthesis, or interpretation. Source-reported GRADE certainty was unavailable in several reviews and ranged from low or very low to higher levels where assessed; therefore, positive findings from reviews with limited or unreported certainty were interpreted cautiously rather than as definitive evidence-based recommendations. In addition, when publication bias was not assessed despite being applicable, the possibility of small-study effects and overestimation of treatment benefit could not be excluded.

### 3.4 Overlap assessment

The final citation matrix contained 170 primary-study occurrences (*N* *=* *170*) across 18 systematic reviews and meta-analyses (*c* *=* *18*). After standardization of author–year labels and deduplication, these occurrences represented 91 unique primary studies (*r* *=* *91*), yielding an overall *CCA* of *5.11%,* corresponding to moderate overlap. However, overlap was substantially greater within intervention-specific clinical questions. Among the six reviews evaluating systemic NSAIDs/analgesics, 51 primary-study occurrences represented 18 unique primary studies (*CCA = 36.67%*; very high overlap). The four PBMT/LLLT reviews contained 27 primary-study occurrences representing 12 unique primary studies (*CCA = 41.67%*; very high overlap). Among the three reviews focused on potassium nitrate/fluoride-based interventions, 40 primary-study occurrences represented 32 unique primary studies (*CCA = 12.50%*; high overlap). Detailed calculations are provided in Supplementary Table S1.

Thus, overlap was not evenly distributed across the evidence base. Although the overall CCA indicated only moderate overlap, substantially greater redundancy was evident within the systemic NSAID/analgesic, PBMT/LLLT, and potassium nitrate/fluoride clusters. Figure 4 presents the intervention-specific citation matrices in three panels: systemic NSAIDs/analgesics (Figure 4A), PBMT/LLLT (Figure 4B), and potassium nitrate/fluoride-focused interventions (Figure 4C). Consequently, agreement among reviews within these intervention categories should not be interpreted as entirely independent replication of the underlying evidence, because several primary trials contributed repeatedly to multiple reviews. Overlap was therefore considered together with AMSTAR-2 assessments, ROBIS judgments, source-reported GRADE ratings, and clinical relevance when formulating the umbrella-level conclusions.

**Figure 4:**
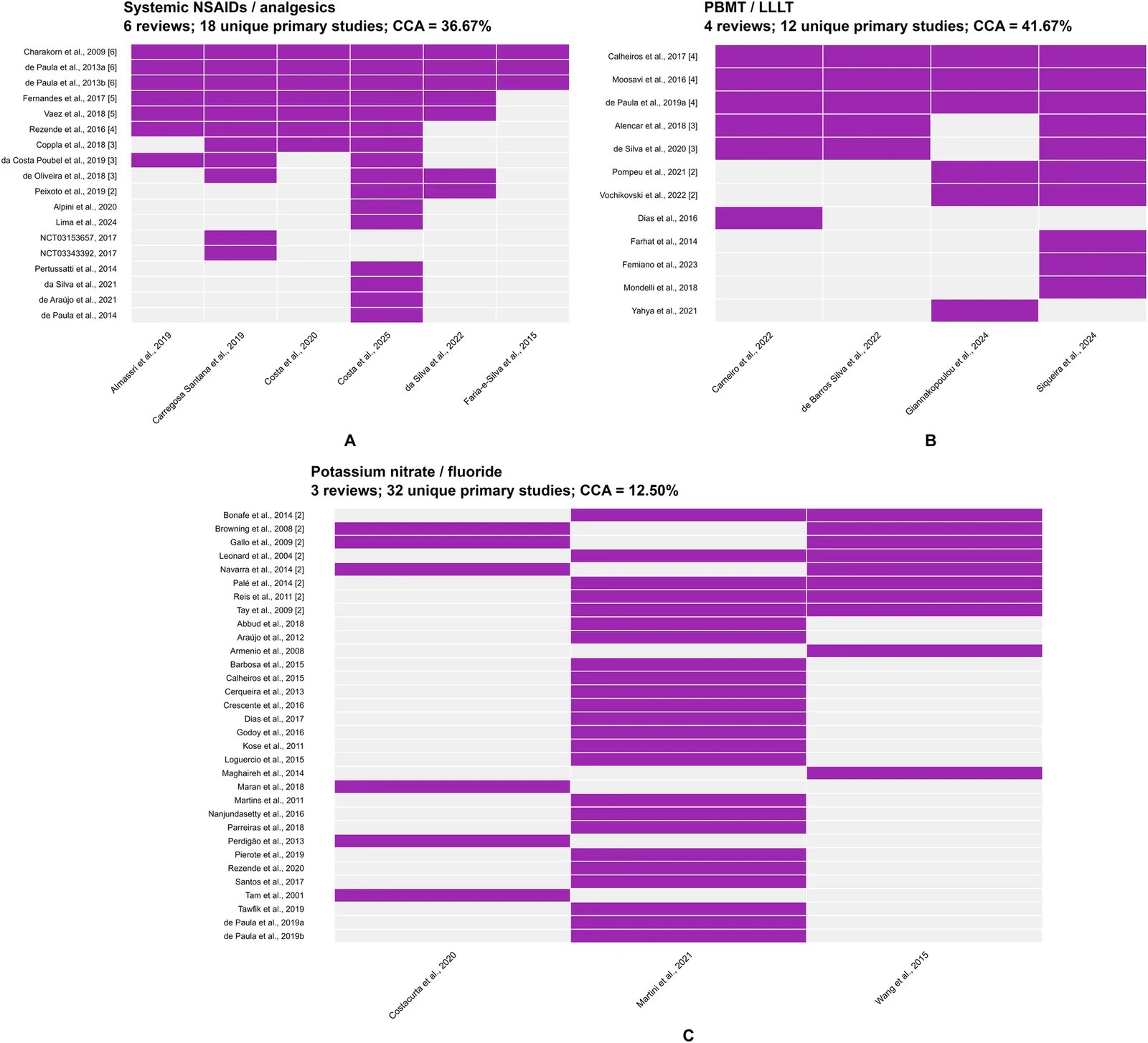
Intervention-specific primary-study overlap across systematic reviews. **(A)** Systemic NSAIDs/analgesics; **(B)** PBMT/LLLT; and **(C)** potassium nitrate/fluoride-focused interventions. Purple cells indicate inclusion of a primary study in the corresponding systematic review, whereas grey cells indicate absence. Numbers in brackets indicate the number of systematic reviews within each intervention category that included the same primary study. The corresponding corrected covered area (CCA) is displayed within each panel.

### 3.5 Synthesis of evidence

The effectiveness of pharmacological and non-pharmacological strategies for preventing or reducing bleaching-induced TS was evaluated across multiple systematic reviews and meta-analyses. Findings were interpreted by intervention type, bleaching setting, peroxide concentration when reported, effect size, confidence intervals, heterogeneity, and clinical meaningfulness.

Among pharmacological approaches, systemic administration of NSAIDs before or during in-office bleaching showed no preventive benefit (2, 5, 14–17). Pooled analyses consistently reported risk ratios for TS incidence close to unity (approximately 0.95–1.10), with confidence intervals crossing the null effect in most reviews, together with only small absolute reductions in TS intensity (<1 VAS unit) and no influence on bleaching efficacy. These findings were consistent across reviews with differing methodological quality and support the conclusion that systemic NSAIDs do not provide clinically important prevention of bleaching-induced TS.

Evidence regarding topical drugs was limited. The review by de Oliveira et al. found that enamel-surface topical drug application, including Otosporin^TM^, ibuprofen plus arginine gel, dipyrone gel, and eugenol nanogel, did not significantly reduce TS at 1 h, 24 h, or 48 h after in-office bleaching, despite low risk of bias in the included split-mouth randomized trials (22). Heterogeneity in drug formulation, molecular size, vehicle, penetration through enamel/dentin, and timing of application limits interpretation and highlights a possible vehicle effect.

Potassium nitrate was the most frequently investigated desensitizing agent (1, 8, 18–20). Wang et al. reported a significant reduction in TS risk (OR = 0.45; 95% CI, 0.28–0.73) (18), whereas later reviews reported smaller or non-significant incidence effects, including potassium nitrate incorporated into bleaching gels (1, 19, 20). Martini et al. reported a modest reduction in TS risk (RR = 0.88; 95% CI, 0.78–0.98) and intensity (VAS MD = −0.77; 95% CI, −1.34 to −0.19). The reduction in intensity represents a small absolute effect, and its clinical perceptibility in patients experiencing bleaching-induced TS remains uncertain (1). Fluoride-containing formulations were usually evaluated in combination with potassium nitrate or other desensitizing agents, making it difficult to isolate the independent effect of fluoride. Calcium-related strategies, including CPP-ACP/ACP, hydroxyapatite, tricalcium phosphate, and calcium sodium phosphosilicate/bioglass, showed small effects mainly on TS intensity rather than incidence, with low to very low source-reported GRADE certainty (6).

Non-pharmacological interventions were primarily focused on PBMT. PBMT-focused reviews generally favored reductions in TS intensity, but the strength of inference varied because protocols differed in wavelength (approximately 660–900 nm), power, energy density, number of irradiation points, and timing of application. For example, Carneiro et al. (10) could not perform meta-analysis because of high heterogeneity (I^2^ = 87%), whereas de Oliveira et al. (22) reported low heterogeneity for topical-drug outcomes (I^2^ = 0%–10%) but no clinical benefit. Network meta-analyses addressing bleaching protocols or light activation were treated as contextual evidence only, because their rankings relate to protocol optimization rather than active desensitizing treatment; no SUCRA-based hierarchy was therefore integrated into the main intervention synthesis. Importantly, active desensitizing interventions generally did not negatively affect bleaching efficacy.

Overall, the evidence suggests that active interventions are more likely to reduce TS intensity than to prevent its occurrence. Confidence in positive findings was lower when source reviews reported low or very low GRADE certainty, high ROBIS risk of bias, substantial unexplained heterogeneity, or other important methodological limitations.

### 3.6 Quality-based sensitivity interpretation

The qualitative quality-based sensitivity interpretation did not involve exclusion or numerical weighting of any eligible systematic review. Instead, the findings from reviews with fewer methodological concerns were compared with the complete evidence base to assess the robustness of the intervention-level conclusions.

The conclusion that systemic NSAIDs provide no meaningful preventive benefit remained unchanged because the direction of effect was consistent across reviews with differing methodological quality and risk-of-bias profiles. For potassium-nitrate-based interventions, the overall interpretation also remained unchanged: the evidence suggested small reductions in TS intensity, while effects on incidence were less consistent. Evidence for calcium-based interventions was interpreted more cautiously because the reported effects were small and the source review reported low or very low certainty for relevant outcomes. PBMT remained a potentially beneficial intervention, particularly for reducing early post-bleaching TS, but confidence in the magnitude and reproducibility of this effect was limited by substantial clinical heterogeneity in irradiation protocols, variability in bleaching and co-intervention regimens, inconsistent methodological quality across reviews, and considerable overlap of the underlying primary studies. Taken together, these methodological and evidentiary limitations did not reverse the direction of the principal intervention-level conclusions but reduced confidence in the strength and independence of some positive findings.

## 4 Discussion

### 4.1 Interpretation of findings

This umbrella review synthesizes evidence from multiple systematic reviews and meta-analyses evaluating pharmacological and non-pharmacological strategies for preventing or reducing bleaching-induced TS. In general, TS remains a common early adverse effect of vital bleaching, typically peaking within the first 24–72 h after treatment. Most interventions demonstrated modest reductions in TS intensity, whereas consistent reductions in TS incidence were uncommon.

Topical desensitizing agents based on potassium nitrate showed statistically significant but clinically modest reductions in sensitivity outcomes (1). The observed reductions in sensitivity intensity were generally small in absolute magnitude. The approximately 1-point benchmark on a 10-point VAS reported in broader acute-pain literature (23, 24) has not been treated in this review as a validated MCID specific to bleaching-induced TS. Therefore, the clinical perceptibility of these small reductions remains uncertain. Protocol characteristics also influence TS risk: higher peroxide concentrations (4), longer exposure times (25), tray design (26), and whether the protocol is at-home or in-office may modify both incidence and intensity (3).

The variable effectiveness of interventions can be explained by the biological mechanisms underlying TS. Hydrogen peroxide can diffuse through enamel and dentin into the pulp chamber where reactive oxygen species may induce oxidative stress, inflammatory mediator release, and transient pulpal irritation (27, 28). Systemic NSAIDs aim to reduce inflammation through inhibition of cyclooxygenase enzymes and prostaglandin synthesis; however, the transient and localized nature of bleaching-induced inflammation may explain their limited clinical effectiveness (29).

In contrast, topical desensitizing agents act directly at the dentin surface. Potassium nitrate may stabilize pulpal nerve membranes, while fluoride and calcium-based compounds reduce dentinal fluid movement through tubule occlusion. These localized mechanisms may explain the modest reductions in TS intensity reported in clinical studies (18).

Among non-pharmacological approaches, PBMT has shown promising results, with reductions in TS intensity reported across systematic reviews (9–12). However, treatment protocols varied substantially in wavelength, power, fluence, irradiation time, application site, timing relative to bleaching, and number of applications. In addition, several protocol characteristics were incompletely reported by the source reviews, and the underlying primary evidence showed substantial overlap across reviews. Consequently, although PBMT may reduce early post-bleaching TS, the current evidence does not support a strong recommendation for any specific wavelength, dose, irradiation site, or treatment schedule.

### 4.2 Clinical implications

Bleaching-induced TS is typically transient and most intense during the first days after treatment. Clinicians should therefore focus on early management strategies and patient counseling. Potassium-nitrate-based desensitizers may provide modest relief for patients concerned about sensitivity, but the absolute benefit is small and its patient-perceived clinical importance remains uncertain (1). Baseline sensitivity, gingival recession, bleaching setting, peroxide concentration, and tray type should be considered when selecting a preventive strategy.

Adjustments to bleaching protocols—such as reduced exposure time, neutral-pH formulations, careful tray adaptation, and avoidance of excessive gel volume—may help minimize sensitivity without compromising whitening outcomes (30). PBMT may be considered where appropriate equipment and expertise are available; however, substantial protocol heterogeneity and incomplete reporting of treatment parameters currently preclude recommendation of a standardized clinical regimen.

### 4.3 Strengths and limitations

This umbrella review provides a structured synthesis of evidence derived from multiple systematic reviews and meta-analyses. Its strengths include assessment of methodological quality using AMSTAR-2, separate assessment of risk of bias using ROBIS, extraction of reported GRADE certainty, and formal evaluation of primary-study overlap using the CCA approach. These methods allowed conclusions to be interpreted according to both statistical findings and confidence in the underlying reviews.

However, several limitations should be considered. Substantial heterogeneity existed across primary studies in bleaching protocols, peroxide concentrations, tray systems, baseline sensitivity, desensitizing agents, vehicles, application timing, and outcome assessment methods. Several reviews did not evaluate publication bias, and many reported low or very low certainty because of risk of bias, inconsistency, and imprecision. Although the overall CCA indicated moderate overlap (5.11%), overlap was unevenly distributed across intervention categories. Intervention-specific analyses demonstrated very high overlap among reviews of systemic NSAIDs/analgesics (36.67%) and PBMT (41.67%), and high overlap among potassium nitrate/fluoride-focused reviews (12.50%). Therefore, apparent consistency across multiple reviews within these intervention categories should be interpreted cautiously, because several reviews relied on overlapping sets of primary studies.

An additional limitation concerns the incomplete and inconsistent reporting of PBMT treatment parameters across the source systematic reviews. Although wavelength and some dosimetric characteristics were frequently reported, energy per point, irradiation time, number and location of irradiation points, and exact treatment schedules were not consistently available. This limits reproducibility, comparison across protocols, and identification of an optimal clinical PBMT regimen.

### 4.4 Future research

Future studies should prioritize standardized TS outcomes, reporting of baseline sensitivity and gingival recession, peroxide concentration, tray type, intervention vehicle, timing of application, and patient-perceived clinical relevance. PBMT studies should additionally report and systematically evaluate key treatment parameters, including wavelength, output power, energy per point, fluence, irradiation time, irradiation sites, timing relative to bleaching, and number of treatment applications. Future systematic reviews should also report heterogeneity statistics I^2^, confidence intervals, publication-bias assessments, GRADE certainty, and sensitivity analyses that account for low-quality or high-risk-of-bias evidence.

## 5 Conclusions

Systemic NSAIDs do not appear to provide a meaningful preventive benefit for bleaching-induced tooth sensitivity, and this conclusion was consistent across multiple reviews. Potassium-nitrate-based interventions may provide small reductions in TS intensity, although effects on TS incidence remain inconsistent. Evidence for calcium-based interventions remains uncertain because reported benefits are small and source-reported certainty is generally low or very low. PBMT may reduce early post-bleaching TS, but the available evidence is limited by substantial heterogeneity in irradiation protocols, incomplete reporting of treatment parameters, methodological variability, and considerable overlap of the underlying primary studies. Accordingly, no specific PBMT protocol can currently be recommended. Overall, the evidence supports greater confidence in the lack of benefit of systemic NSAIDs than in the effectiveness of any active desensitizing intervention, and potentially beneficial approaches should be interpreted according to their differing levels of evidentiary support.

## Data Availability

All data analyzed in this study are derived from previously published articles. The dataset generated during the current study, including extracted data and methodological assessments, is available in the Open Science Framework repository: https://osf.io/9s8h7.
