# Supplementary material for: Interventions for bleaching-induced tooth sensitivity: an umbrella review
Source: Front Oral Health. 2026 Sep 21;7:1927686. doi: 10.3389/froh.2026.1927686 (PMC13634835; doi:10.3389/froh.2026.1927686)
Supplement: Supplementary file 1 [file Table1.docx]

Supplementary Table S1. Overall and intervention-specific corrected covered area (CCA) across the included systematic reviews and meta-analyses

| Evidence cluster | Systematic reviews included in cluster | Systematic reviews (*c*) | Primary-study occurrences (*N*) | Unique primary studies (r) | *CCA* (%) | Interpretation |
| --- | --- | --- | --- | --- | --- | --- |
| Systemic NSAIDs/analgesics | Almassri et al., 2019; Faria-e-Silva et al., 2015; Carregosa Santana et al., 2019; Costa et al., 2020; da Silva et al., 2022; Costa et al., 2025 | 6 | 51 | 18 | 36.67 | Very high |
| PBMT | Carneiro et al., 2022; de Barros Silva et al., 2022; Giannakopoulou et al., 2024; Siqueira et al., 2024 | 4 | 27 | 12 | 41.67 | Very high |
| Potassium nitrate/fluoride-focused interventions | Wang et al., 2015; Costacurta et al., 2020; Martini et al., 2021 | 3 | 40 | 32 | 12.50 | High |
| Calcium-based interventions | Favoreto et al., 2023 | 1 | — | — | Not applicable | Single dedicated review |
| Overall evidence base | All 18 included systematic reviews | 18 | 170 | 91 | 5.11 | Moderate |

Note: CCA = [(N − r)/(r × c − r)] × 100%, where N represents the total number of primary-study occurrences, r the number of unique primary studies, and c the number of systematic reviews. CCA was interpreted as slight (0–5%), moderate (>5–10%), high (>10–15%), or very high (>15%). Intervention-specific CCA analyses were performed only for intervention categories represented by at least two systematic reviews addressing the same clinical question. Reviews evaluating broader or mixed desensitizing interventions were included in the overall CCA but were not assigned to a separate intervention-specific cluster. CCA was not calculated for calcium-based interventions because this category was represented by only one dedicated systematic review (c = 1).

Supplementary Table S2. PBMT parameters reported in the source systematic reviews

| Primary study | Source review(s) | Wavelength (nm) | Power | Energy per point | Fluence | Irradiation time | No./location of irradiation points | Timing relative to bleaching | PBMT sessions/applications |
| --- | --- | --- | --- | --- | --- | --- | --- | --- | --- |
| Moosavi et al., 2016 | de Barros Silva 2022; Carneiro 2022; Giannakopoulou 2024; Siqueira 2024 | 660 (red) and 810 (infrared) | 200 mW | 3 J/tooth | 12 J/cm² | 15 s | Cervical area of each tooth; 5 × 5-mm aperture | After bleaching | Exact total schedule inconsistently described across source reviews |
| Calheiros et al., 2017 | de Barros Silva 2022; Carneiro 2022; Giannakopoulou 2024; Siqueira 2024 | 780 | 40 mW | 0.4 J/point | 10 J/cm² | 10 s/point | 2 points/tooth: middle of crown and periapical region; direct contact | Before, after, or both before and after bleaching, depending on study arm | 2 bleaching sessions; PBMT before, after, or both at each session |
| Alencar et al., 2018 | de Barros Silva 2022; Carneiro 2022; Siqueira 2024 | 810 | 100 mW | NR; total 3.4 J/session reported | 60 J/cm²/point; total 120 J/cm²/session | 16 s/point | 2 points: central cervical and medial regions of incisors, canines, and premolars; direct contact | Before bleaching | PBMT applied in association with the bleaching sessions; 4 weekly bleaching sessions reported |
| de Paula et al., 2019 | de Barros Silva 2022; Carneiro 2022; Giannakopoulou 2024; Siqueira 2024 | 808 | 100 mW | 1.7 J/point | 60 J/cm²/point; total 120 J/cm²/session | 16 s/point | 2 points: apical and cervical regions on buccal surfaces of incisors, canines, and premolars | After bleaching | 3 bleaching sessions; 3 PBMT applications reported |
| de Silva et al., 2020 | de Barros Silva 2022; Carneiro 2022; Siqueira 2024 | 808 | 100 mW | 1.7 J/point | 60 J/cm²/point; total 120 J/cm²/session | 16 s/point | 2 points: apical and central regions on buccal surfaces of incisors, canines, and premolars | Before bleaching | 3 weekly bleaching sessions; PBMT applied before bleaching |
| Dias et al., 2016 | Carneiro 2022 | 780 | 40 mW | NR | 10 J/cm² | 10 s | NR | Immediately after bleaching | NR |
| Pompeu et al., 2021 | Giannakopoulou 2024; Siqueira 2024 | 808 | 100 mW | 1.7 J reported; per-point basis not specified in review | 60 J/cm² | NR | NR | Post-bleaching | 3 bleaching sessions; source review reports 1 laser application, exact schedule not further specified |
| Yahya et al., 2021 | Giannakopoulou 2024 | 660–900 | 90 mW | NR | NR | NR | NR | Post-bleaching desensitizing intervention; exact timing NR | 1 bleaching session; 1 laser application |
| Vochikovski et al., 2022 | Giannakopoulou 2024; Siqueira 2024 | 808 | 100 mW | NR | 100 J/cm² | NR | NR | After each bleaching session | 2 bleaching sessions, 1 week apart; PBMT after each session |
| Farhat et al., 2014 | Siqueira 2024 | Blue LEDs 425–480 + infrared laser 810 | LEDs 300 mW; IR lasers 200 mW | NR | 300 mW/cm² per tooth* | 1 min activation ×3, with 2-min resting intervals | Hybrid light source; exact irradiation points NR | During bleaching | NR |
| Femiano et al., 2023 | Siqueira 2024 | 810 | 0.5 W | NR | 15 J/cm² | 30 s | NR | Before bleaching | NR |
| Mondelli et al., 2018 | Siqueira 2024 | Blue LEDs 470 + infrared diode lasers 810 | LEDs 350 mW/cm²; IR lasers 200 mW/cm² | 25 J reported | NR | 30 s | Hybrid source; exact points NR | After bleaching | NR |

**Note:** PBMT, photobiomodulation therapy; LLLT, low-level laser therapy; PBM, photobiomodulation; NR, not reported; IR, infrared; LED, light-emitting diode; HP, hydrogen peroxide; J, joule; J/cm², joules per square centimeter; mW, milliwatt; W, watt; nm, nanometer; s, second; VAS, visual analog scale; VRS, verbal rating scale. PBMT parameters were extracted only when reported by the included systematic reviews and were not supplemented from the original primary studies. Because several primary trials were included in more than one source review, each unique primary study is presented once and the contributing source reviews are identified. Where reporting differed or was incomplete across reviews, this is indicated rather than inferred.
